# BeeDoctor, a Versatile MLPA-Based Diagnostic Tool for Screening Bee Viruses

**DOI:** 10.1371/journal.pone.0047953

**Published:** 2012-10-29

**Authors:** Lina De Smet, Jorgen Ravoet, Joachim R. de Miranda, Tom Wenseleers, Matthias Y. Mueller, Robin F. A. Moritz, Dirk C. de Graaf

**Affiliations:** 1 Laboratory of Zoophysiology, Department of Physiology, Ghent University, Ghent, Belgium; 2 Department of Ecology, Swedish University of Agricultural Sciences, Uppsala, Sweden; 3 Laboratory of Socioecology and Social Evolution, KU Leuven, Leuven, Belgium; 4 Institüt für Biologie, Martin Luther Universität Halle-Wittenberg, Halle/Saale, Germany; Central China Normal University, China

## Abstract

The long-term decline of managed honeybee hives in the world has drawn significant attention to the scientific community and bee-keeping industry. A high pathogen load is believed to play a crucial role in this phenomenon, with the bee viruses being key players. Most of the currently characterized honeybee viruses (around twenty) are positive stranded RNA viruses. Techniques based on RNA signatures are widely used to determine the viral load in honeybee colonies. High throughput screening for viral loads necessitates the development of a multiplex polymerase chain reaction approach in which different viruses can be targeted simultaneously. A new multiparameter assay, called “BeeDoctor”, was developed based on multiplex-ligation probe dependent amplification (MLPA) technology. This assay detects 10 honeybee viruses in one reaction. “BeeDoctor” is also able to screen selectively for either the positive strand of the targeted RNA bee viruses or the negative strand, which is indicative for active viral replication. Due to its sensitivity and specificity, the MLPA assay is a useful tool for rapid diagnosis, pathogen characterization, and epidemiology of viruses in honeybee populations. “BeeDoctor” was used for screening 363 samples from apiaries located throughout Flanders; the northern half of Belgium. Using the “BeeDoctor”, virus infections were detected in almost eighty percent of the colonies, with deformed wing virus by far the most frequently detected virus and multiple virus infections were found in 26 percent of the colonies.

## Introduction

Honeybees provide both honey and key pollination services to much of the world [1]–[3]. Overall pollinator populations, including wild and feral honeybee (*Apis mellifera*) populations, have been declining consistently worldwide due to a variety of causes [4]. Annual losses of managed honeybee populations have also increased significantly during the last decades, highlighted by the recent dramatic mass colony losses in the USA due to Colony Collapse Disorder (CCD; [5]), as well as increased winter colony losses and reduced honeybee and queen vitality, largely due to pathogens and parasites [6], including mites (*Varroa destructor*, *Acarapis woodi*, *Tropilaelaps* sp.), microsporidia (*Nosema* spp.), fungi (chalkbrood; *Ascosphaera apis*), bacteria (American foulbrood (*Paenibacillus larvae*), European foulbrood (*Melissococcus plutonius*)), viruses, and pests (large wax moth *Galleria melonella*, small hive beetle *Aethina tumidae*) [7], [8].

Recently the role of pathogenic viruses gained particular interest since they have been suspected to be important drivers of colony declines. Honeybees are host to between 12–20 viruses, depending on classification [9], most of which are positive strand RNA viruses belonging to *Picornavirales* [10]. Black queen cell virus (BQCV) [11] and the acute bee paralysis virus complex [12] including Acute bee paralysis virus (ABPV [13]), Kashmir bee virus (KBV; [14]) and Israeli acute paralysis virus (IAPV [15]), belong to the Dicistroviridae family. Deformed wing virus (DWV) [16] and close relatives Varroa destructor virus-1 (VDV-1; [17]) and Kakugo virus (KV; [18]), Slow bee paralysis virus (SBPV) [19] and Sacbrood virus (SBV) [20] belong to the Iflaviridae family while chronic bee paralysis virus (CBPV) [21] and relatives are putative Nodaviridae [22]. Several other known viruses, including bee virus X and Y (BVX; BVY), cloudy wing virus (CWV), *Apis mellifera* filamentous virus (*Am*FV), Apis iridescent virus (AIV); Arkansas bee virus (ABV) and Berkeley bee virus (BBPV) remain to be fully characterized molecularly [23], while on the other hand next-generation sequencing techniques have identified several novel viruses (some of which may be the same as the uncharacterized viruses named above) and microbes [22], [24], [25] through which the honeybee pathosphere has been expanded and this is likely to continue in the near future. Symptoms associated with specific viruses include wing deformities (DWV), hairless, dark, shiny bees (CBPV), swollen yellow larvae and/or dark-brown larva carcasses in the cells of worker-bees (SBV) or in queen cells (BQCV). Many virus infections also cause behavioral aberrations, such as shivering, paralysis, disorientation, aggression or altered foraging preferences or changes in brood care [26]. The appearance of symptoms generally requires high virus titres; the result of close transmission within the colony. Most commonly however, viral infections in honeybees are low-medium titre and thus asymptomatic. Accurate diagnoses of such asymptomatic virus infections therefore requires molecular techniques.

The detection of viral infections in honeybees is increasingly based on the detection of specific viral genomic nucleic acids. Since most (honeybee) viruses have RNA genomes, this means the detection of virus-specific RNA signatures. The most widely used method is reverse transcriptase quantitative PCR (RT-qPCR). Many individual RT-(q)PCR protocols have been described for the detection of specific honeybee viruses (review [27]) as well as several multiplex RT-PCR approaches [28]–[30]. Multiplex detection approaches, where several targets are detected and quantified simultaneously, are increasingly important, both for reducing costs and more importantly for studying the complex interactions between different targets, which can include important host genes as well as RNA-based pathogens. However, the optimization of multiplex RT-PCR can pose several difficulties, including poor sensitivity and specificity, and/or preferential amplification of certain specific targets [31]. Furthermore, real-time multiplex assays are mostly restricted to detection of up to four or five targets in a reaction, depending on the number of channels available in the used PCR machine. Multiplex Ligation-dependent Probe Amplification (MLPA) is an amplification technique that allows simultaneous detection of up to 45 different targets with the use of a single primer set [32]. MLPA is based on the ligation of two adjacent oligonucleotides hybridizing next to each other on a single-stranded target template. The ligated oligonucleotides (‘probe’) serve as template for PCR-based amplification and detection. Apart from virus-specific sequences, each oligonucleotide (‘half-probe’) contains a universal tag, for simultaneous PCR-based amplification of multiple targets with a single PCR primer pair, and a non-specific stuffer fragment for generating controlled size differences between different targets. The different targets are identified by size using electrophoresis. Because honeybee viruses are RNA viruses, a reverse transcription step is added prior to MLPA (RT-MLPA). MLPA and RT-MLPA assays have recently been developed for the simultaneous detection of several virus species causing central nervous system infections [33]. Another application called RespiFinder™ tests differentially for fifteen respiratory viruses [34]. MLPA is also used for the detection of other pathogens like *Mycobacterium tuberculosis* [35], bacterial species in oral biofilms [36], *Penicillium marneffei* [37] and different opisthorchid liver fluke species [38].

Replication in positive-strand RNA viruses, such as many honeybee viruses, proceeds via the production of a negative-strand intermediate. Strand-specific RT-PCR was first developed for detection of negative-strand RNAs of viruses [39], [40]. However, strand-specific RT-PCR is very sensitive to false-positive results, primarily due to mis-priming and self-priming of the RNA during reverse transcription [41]. These inadequacies have been addressed with a combination of additional steps, primarily by using tagged cDNA primers and purifying the cDNA from residual primer prior to PCR amplification [42], [43]. The RT-MLPA is ideal for strand-specific detection of nucleic acids since it amplifies a probe (rather than the original target) that can only be produced in a strand-specific manner, through ligation of two oligonucleotide half-probes hybridizing to a complementary cDNA target. The ligase-65 used to ligate the two half-probes to each other is not active on RNA-DNA hybrids, thus avoiding possible false-positive results due to ligation of the half-probes that hybridize directly on target RNA of the same polarity as the cDNA to the opposite strand.

Our report here shows the application of RT-MLPA for simultaneously detecting 10 targeted honeybee viruses. Two MLPA probe sets were developed which are able to detect selectively the positive strand RNA or the replicative negative strand RNA intermediate. The possibility to screen easily for replication will be valuable for studying virus replication and pathogenesis in naturally infected hosts. Because of its high sensitivity and specificity, the RT-MLPA assay is also a useful tool for prompt diagnosis and epidemiological studies of viruses in honeybee populations. Al last, we used this newly developed method in an epidemiological survey of honeybee viruses based on adult bee samples collected in Flanders during the summer of 2011.

## Materials and Methods

### Samples

All Flemish beekeepers were invited to participate in an epidemiological survey for virus screening; 170 beekeepers accepted the invitation and submitted a total of 363 samples of 30 adult bees, collected in July 2011 at the entrance of colonies that seemed healthy and that were not (yet) treated against *Varroa*. Generally, each beekeeper sent two samples from their apiary. The bees were immediately frozen at −20°C until their shipment to the laboratory, where upon arrival they were stored at −80°C until RNA extraction. Excess bees were archived for long-term −80°C storage.

### Nucleic acid extraction

To detect the positive strand viral RNA was isolated by using the QiaAmp Viral RNA mini kit (Qiagen). Individual whole adult bees were ground in a mortar in 1 ml ice-cold PBS per bee. The extract was centrifuged at 14 000× g and RNA was extracted from 140 µl of the liquid supernatant according to the manufacturer's instructions, eluting the RNA in a final volume of 50 µl. In the negative strand detection mode the total RNA was isolated using the RNeasy lipid tissue mini kit (Qiagen) starting from one complete honeybee. The tissue was homogenized by mechanical agitation in a TissueLyser (Precellys) for 90 sec at 30 Hz, in the presence of a pair stainless steel beads and 1 ml QIAzol lysis reagent. The total RNA was isolated according to the recommendation of the manufacturer's protocol, eluting the RNA in a final volume of 50 µl.

For the Flanders virus survey, 10 bees per colony were homogenized in a total of 5 ml PBS by mechanical agitation in a TissueLyser for 90 sec at 30 Hz, in the presence of glass beads. The extract was centrifuged at 14 000× g and RNA was extracted from 140 µl of the liquid supernatant using the QiaAmp Viral RNA mini kit according to the manufacturer's instructions, as outlined above.

### Probe design

MLPA probes and RT-primers were designed for 6 virus targets, covering the 10 most common honeybee viruses, and for two honeybee internal reference genes; β-actin and ribosomal protein 8 (RPL8), as positive controls for the quality of the RNA samples. For each virus or virus-complex a pair of probes was designed following the guidelines described in the manual “Design synthetic MLPA probes” (MRC Holland, Amsterdam, The Netherlands). All probe pairs contain the same universal binding sites for the reverse PCR primer on the right probe oligo (RPO) and for the forward PCR primer on the left probe oligo (LPO). The probes were designed using the AlleleID ® software (PREMIER Biosoft) against the most conserved regions within each virus or virus family as determined by aligning all available gene sequences in the GenBank using Clustal X program. An additional selection criterium was the absence of mismatches within 5 nucleotides from the ligation site. The uniqueness of our selected probe sequences was inspected by BLAST analysis at the NCBI website (www.ncbi.nlm.nih.gov). The primers for cDNA synthesis were positioned immediately adjacent to the MLPA probe, with no more than 15 nucleotides between the last nucleotide of the RT primer and the first nucleotide of the probe sequence, and with a maximum overlap of 7 nucleotides. The RT primers were designed with Primer 3 software (http://primer3.sourceforge.net). All primers and probes were synthesized by Integrated DNA Technologies (Leuven, België). The RPO should be 5′ phosphorylated and synthesized with ‘ultramers’ quality. The RTprimers, MLPA half-probes (LPO and RPO) and PCR amplification primers used in the experiments are listed in Table 1.

**Table 1:** Primers and half-probes used for detecting either the positive or negative (replicative) strand of different honeybee viruses and virus species complexes through RT-MLPA.

VIRUS	STRAND	FUNCTION	SEQUENCE (5′-3′)	SIZE (bp)
CBPV	**+ (pos)**	(−)cDNA	GCCCCGATCATATAAGCAAA	88
		(+)MLPA-LPO	gggttccctaagggttggaCCGTAGCTGTTTCTGCTGCGGT	
		(+)MLPA-RPO	^P-^ ACTCAGCTCAGCTCGACGCTCAGAtctagattggatcttgctggcac	
	**− (neg)**	(+)cDNA	GAACATCCGGAACAGACGAT	88
		(−)MLPA-LPO	gggttccctaagggttggaTCTGAGCGTCGAGCTGAGCTGAGT	
		(−)MLPA-RPO	^P-^ ACCGCAGCAGAAACAGCTACGGtctagattggatcttgctggcac	
DWV/KV	**+ (pos)**	(−)cDNA	TCACATTGATCCCAATAATCAGA	95
VDV-1		(+)MLPA-LPO	gggttccctaagggttggaTGACCGATTCTTTATGCAGCGAGCTCT	
		(+)MLPA-RPO	^P-^ TACGTGCGAGTCGTACTCCTGTGACAtctagattggatcttgctggcac	
	**− (neg)**	(+)cDNA	GTGTGGTGCATCTGGAATTG	95
		(−)MLPA-LPO	gggttccctaagggttggaGTTGTCACAGGAGTACGACTCGCA	
		(−)MLPA-RPO	^P-^ CGTAAGAGCTCGCTGCATAAAGAATCGGTtctagattggatcttgctggcac	
ABPV	**+ (pos)**	(−)cDNA (ABPV)	CAATGTGGTCAATGAGTACGG	104
KBV		(−)cDNA (KBV&IAPV)	TCAATGTTGTCAATGAGAACGG	
IAPV		(+)MLPA-LPO	gggttccctaagggttggaCTCACTTCATCGGCTCGGAGCATGGATGAT	
		(+)MLPA-RPO	^P-^ ACGCACAGTATTATTCAGTTTTTACAACGCCCtctagattggatcttgctggcac	
	**− (neg)**	(+)cDNA	TGAAACGGAACAAATCACCA	104
		(−)MLPA-LPO	gggttccctaagggttggaCGAGCCGATGAAGTGTCTTGAGCCATGG	
		(−)MLPA-RPO	^P-^ GGGTATTGATCCTATTTGGAGTTTCCACATCATGtctagattggatcttgctggcac	
BQCV	**+ (pos)**	(−)cDNA	CGGGCCTCGGATAATTAGA	122
		(+)MLPA-LPO	gggttccctaagggttggaCTTCATGTTGGAGACCAGGTTTGTTTGCCGACTTACGGAA	
		(+)MLPA-RPO	^P-^ TGTCGTTAAACTCTAGGCTTTCCGGATGGCTTCTTCATGGtctagattggatcttgctggcac	
	**− (neg)**	(+)cDNA	TTAAAAGCCCCGTATGCTTG	122
		(−)MLPA-LPO	gggttccctaagggttggaTCAGCGCAACAGAAGCCATCCGGAAAGCCTAGAGTTTAACG	
		(−)MLPA-RPO	^P-^ ACATTCCGTAAGTCGGCAAACAAACCTGCCTTATCTGGTtctagattggatcttgctggcac	
SBPV	**+ (pos)**	(−)cDNA	CGCAAACACGACGAATTTTA	131
	**+ (pos)**	(+)MLPA-LPO	gggttccctaagggttggaCGTTCAATGGTCGAGATAGAAGCCACAGTAGAAGTATTACGCGCT	
		(+)MLPA-RPO	^P-^ TCTTGTGTTTTGGCTTATGGGCGTGGGCCTGATCTTCATTCAGCtctagattggatcttgctggcac	
	**− (neg)**	(+)cDNA	GGTGTCATAAACAGAATGACGAG	131
		(−)MLPA-LPO	gggttccctaagggttggaTCAGCGCAACACTCAGGCCCACGCCCATAAGCCAAAACACAAGAA	
		(−)MLPA-RPO	^P-^ GCGCGTAATACTTCTACTGTGGCTTCTATCTCGCCTTATCTGGTtctagattggatcttgctggcac	
SBV	**+ (pos)**	(−)cDNA	TGGACATTTCGGTGTAGTGG	140
		(+)MLPA-LPO	gggttccctaagggttggaCGTTGATCCAATGGTCAGTGGACTCTTATACCGATTTGTTTAATGGTTGG	
		(+)MLPA-RPO	^P-^ GTTTCTGGTATGTTTGTTGACAAGAACGTCCACCTTCAGCCATTCAGCtctagattggatcttgctggcac	
	**− (neg)**	(+)cDNA	CCTTACCTCTAGTAAGAAGACATTTGA	140
		(−)MLPA-LPO	gggttccctaagggttggaTAAAAAACTACCGTGTAGTGGACGTTCTTGTCAACAAACATACCAGAAA	
		(−)MLPA-RPO	^P-^ CCCAACCATTAAACAAATCGGTATAAGAGTCCACTGAAAAGTCGGTGGAtctagattggatcttgctggcac	
β-Actin	**+ (pos)**	(−)cDNA	TTTCATGGTGGATGGTGCTA	182
		(+)MLPA-LPO	gggttccctaagggttggaGCAGGAAGTCGTTACCACCTGGCCCACGGAGCCAATTTCTCATGCTTGCCAACACTGTCCTTTCTGGAGGT	
		(+)MLPA-RPO	^P-^ ACCACCATGTATCCTGGAATCGCGAAAACGTGGTGTACCGGCTGTCTGGTATGTATGAGTTTGTGGTGAtctagattggatcttgctggcac	
RPL8	**+ (pos)**	(−)cDNA	TGCTTTACCAATATGTTGATGATT	168
		(+)MLPA-LPO	gggttccctaagggttggaTCGGTGAGACGTGGGAGGCGAAAATTGGCGTGTTGGCCTAAGGTTCGTGGTGTTGCTATGAAC	
		(+)MLPA-RPO	^P-^ CCTGTTGAACATCCACACGGTGGTGGTAATCATAACGTCCGGATGCTGAAGTGATGGCAGAGCtctagattggatcttgctggcac	
**PCR**		PCR-Forward	gggttccctaagggttgga	n.a.
		PCR-Reverse	gtgccagcaagatccaatctaga	

The PCR sequence tags on each halfprobe are in lower-case letters, the non-specific stuffer sequences (for generating PCR products with pre-determined sizes) are shown in upper-case letters and the target-specific sequences are shown in underlined upper-case letters. Each RPO probe is 5′-phosphorylated (indicated by ^P-^)to permit ligation of the 5′ end of the RPO to the 3′ end of the LPO.

### MLPA reaction

MLPA analysis was performed essentially as described earlier [32]. All the MLPA reagents were obtained from MRC-Holland (Amsterdam, the Netherlands). All reaction steps were performed in a thermocycler with heated lid (105°C) using 0.2 ml thin-walled PCR tubes. Briefly, 1 µl RNA (between 10–500 ng total RNA), unless otherwise mentioned, was reverse transcribed using 30 U MMLV reverse transcriptase (Promega) in a 6 µl reaction with 0.5 µl RT primer/dNTP mix consisting of 5 pmol/µl of the RT primer for each target virus and 5 mM dNTPs. After 1 min 80°C and 5 min 45°C the reverse transcriptase was added to reaction and was incubated for 15 min at 37°C and deactivated for 2 min at 98°C. A probe mix containing all half-probe oligos for either positive–strand detection or negative-strand detection was prepared containing 1.33 fmol/µl of each oligo. A mixture of 1.5 µl of the probe-mix and 1,5 µl of MLPA buffer was added to each RT reaction and hybridized overnight at 60°C after 1 min denaturation at 95°C. The hybridized probes were ligated together using the Ligase-65 enzyme in a 40 ul reaction at 54°C for 15 min followed by ligase inactivation at 98°C for 5 min. Subsequently, 10 µl of the ligation reaction was used as template for the PCR reaction, using the universal forward and reverse PCR primers (Table 1) in a total reaction volume of 50 ul. PCR amplification was performed for 35 cycles (30 s–95°C, 30 s–55°C and 1 min–72°C) with a final extension step at 72°C for 20 min.

### Analysis of PCR products

The amplified MLPA products were analyzed on different detection platforms. The MLPA was optimized by analysis 10 µl of the MLPA reaction on 4% high resolution agarose gel electrophoresis. As alternative an aliquot of 10 µl was also analysed via capillary electrophoresis using a High Resolution gel cartridge on a QIAxcel platform (Qiagen, Hilden, Germany).

For the Flanders virus survey study the MLPA reactions were analyzed using 4% high resolution agarose gel electrophoresis.

### Cloning and construction of specific MLPA ladder

Fragments generated by the RT-MLPA reactions were desalted with MSB Spin PCRapace (Invitek) and subsequently cloned into pCR4-TOPO vector from TOPO TA Cloning Kit for sequencing (Invitrogen, USA) according to manufacturer's instructions. The cloned inserts were sequenced on a ABI 3130XL platform using the vector primers. Positive constructs were used as template in a standard PCR reaction to amplify the expected MLPA reaction products. The concentrations of the different products were determined using a nanodrop ND-1000 spectrophotometer (Thermo Scientific). The different products were mixed in equal amounts and 10 ng of each fragment was loaded on a gel as marker to simplify the interpretation of the results.

## Results and Discussion

The MLPA is a popular technique in the human genetics but, according to our knowledge, it has not yet been used in the field of veterinary virology. The multiplexing capacity of the technique is much higher than for PCR assays but far below the capability of microarrays. This medium-scale (1–40-fold) multiplexing ability makes MLPA extremely useful for the simultaneous screening of all honey bee viruses and its simplicity can facilitate widespread acceptance of the technique even in small size molecular laboratories.

**Figure 1:**
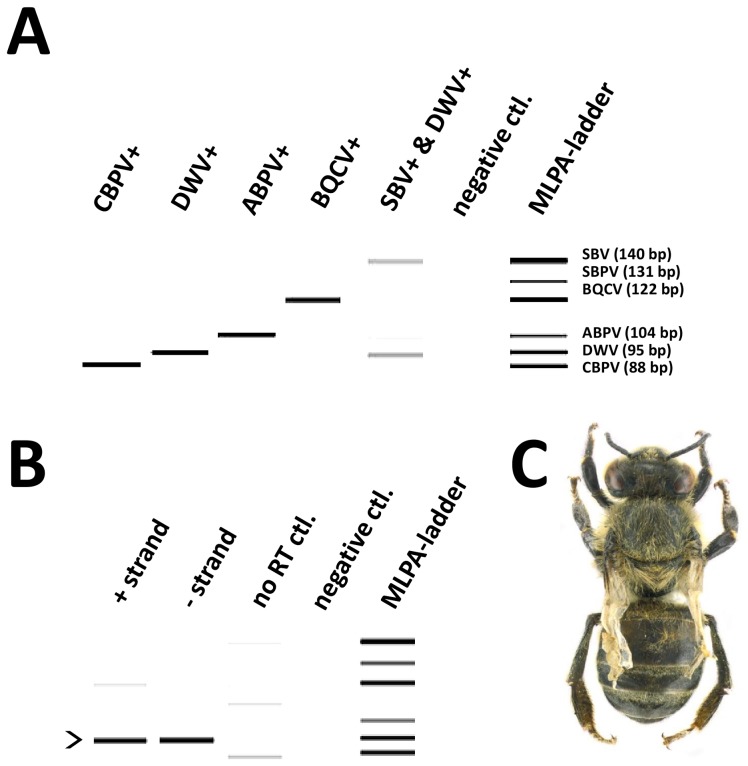
High resolution analysis of MLPa amplicons using the Qiaxcel platform. **A** The result of a MLPA reaction on different samples from which the status was determined by RT-PCR. The status is indicated on top of each lane. The MLPA amplicons were analyzed via capillary electrophoresis using a High Resolution gel cartridge on a QIAxcel platform. Different amplicons of the MLPA ladder are indicated at the right site of panel A. **B** The result of a MLPA reaction on samples with clinical signs of DWV. Both strands, positive and negative strand intermediate could be determined (marked by arrow head). In lane 1 and 3 some weak nonspecific bands are present. In the RT-free control some non-specific products were amplified. **C** A bee with DWV symptoms.

We designed an RT-MLPA approach to detect 6 targets simultaneously covering 10 common honeybee viruses: ABPV, BQCV, IAPV, KBV, DWV, KV, VDV-1, SBPV, SBV, CBPV. We opted for detection by agarose gel electrophoresis, although this part of the protocol can be easily transferred to other platforms, such as capillary electrophoresis or the Agilent bioanalyzer. A spacer in the RT-MLPA probes was included to adjust the final length of the specific RT-MLPA products so that they are separated by 7–9 nucleotide increments, for unambiguous identification of the fragments after electrophoresis (Figure 1A). For DWV, KV and VDV-1 we were not able to design specific probes as their sequences are too related and therefore a consensus probe set was developed for the entire DWV-complex. Similarly, a single consensus probe set was developed for the ABPV-complex of viruses (ABPV, KBV and IAPV). An overview of the probes is given in Table 1.

The diagnostic capacity of the RT-MLPA-assay was tested on (RT-PCR) proven virus positive samples (Figure 1A). All amplicons were cloned and sequenced to confirm their identity. The specificity of the primers and the probes were tested by running all RT primers and MLPA probes in either a monoplex or a multiplex MLPA reaction with different samples. There was no cross-reactivity among the different probes and/or primers.

We constructed an MLPA ladder by mixing equimolar amounts from the different expected RT-MLPA amplicons each corresponding to a specifc virus. These were amplified in a PCR reaction using the different pCR4-cloned RT-MLPA amplicons as templates. The use of this marker greatly facilitates the interpretation of the MLPA results after electrophoresis (Figure 1A).

The sensitivity of the MLPA was tested using synthetic templates for DWV and BQCV. A serial dilution of these templates in total RNA from a non-infected honeybee showed that as few as 1000 copies can be detected with clearly discernible signals. This detection limit is in accordance with the detection limit obtained in other studies [33], [34], [37]. However, too much template in the MLPA reaction can lead to lower detection signals due to inhibition of the PCR reaction [32]. Therefore it is recommended to reduce the initial amount of RNA in the RT-MLPA reaction to 100 ng, in order to minimize the chance of false-negative results.

Well-designed MLPA probes have the ability to discriminate between single-nucleotide polymorphism (SNP) [32]. This means that the viral RT-MLPA probes require a more generic design, as viruses, especially RNA viruses, are generally highly variable due to their very high mutation rates [32]. Therefore, a major concern in the design of the RT-MLPA probes was the compatibility of virus-specific probes with as many known strains of each virus as possible. At the same time, however this generic design should not compromise the specificity of the probe. The probes were therefore positioned in well-conserved regions and no mismatch within 5 nucleotides from the ligation site was tolerated. Although MLPA is widely used to detect SNP, we tested the robustness of our MLPA technique for the presence of mismatches at the ligation site. We synthesized different templates which mimic the DWV target sequences but with either one or two mutations at the ligation point (i.e. the last nucleotide of the LPO and/or the first nucleotide of the RPO) These synthetic templates were used in the MLPA assay, using 5 ng template. Clear positive MLPA results were obtained with the template containing one mutation. The template with two mutations, either side of the ligation site gave very faint signals and the signal was lost completely when the ligation time was shortened from 15 min to 3 min, or when the final amplification step was prematurely aborted at 26 cycles. Other parameters of the MLPA which could influence the detection of mutant templates were also investigated. A positive result was obtained for all mutated templates when the amplification reaction was run for at least 30 cycles. Raising the hybridization temperature up to 66°C did not influence the results. For this particular diagnostic purpose, the detection of honeybee viruses, the insensitivity of the RT-MLPA reaction to nucleotide variations in the target should be seen as an advantage. This makes the diagnostic power of the RT-MLPA approach even stronger.

“BeeDoctor” is optimized to detect various honeybee viruses simultaneously. MLPA is able to multiplex up to 45 targets. Probes used in MLPA usually range between 80 to 400 nucleotides in length. Accurate chemical synthesis of MLPA probes is possible up to a length of approximately 180 nucleotides [44]. As we are using synthetic probes the number of targets in our approach will be limited to 15 to 20. Anyhow the multiplex power of this technique rises far beyond the multiplex power of a real-time quantitative PCR approach which is typically limited to four or five targets depending on the platform used. In order to test the quantitative potential of the assay, we selected two reference genes which were used frequently in honeybee virus research: β-actin and RPL8. A dilution series of DWV synthetic template was spiked into the RNA of non infected honeybees. Unfortunately we observed strong competition between the simultaneously amplified MLPA probes in this case β-actin and DWV probes and hence failed to establish this technique in a quantitative way. However this technique can be widely used in high throughput screening studies. By mixing synthetic templates mimicking the binding sites for DWV en BQCV we could show that the competition problem will not generate false negative results when multiple infected honeybees would be screened. Only the intensities of the generated product are influenced which makes quantification difficult.

All positive-strand RNA viruses replicate and express their genomes through negative-strand RNA intermediates that are used as templates for the production of positive-strand progeny RNAs that are then packaged in new virion particles. Therefore, the presence of negative-strand RNA intermediates is a reliable marker for active virus replication in infected honeybees. It is also a very effective means to distinguish between active infections and the non-infectious, passive presence of virus particles, which is an important epidemiological distinction. RT-MLPA is the ideal technique to selectively detect the positive strand genomic RNA or the negative-strand replicative intermediate RNA, since an amplifiable probe can only be generated in a strand-specific manner, through the ligation of the two half-probes hybridizing next to each other on a single-stranded cDNA target. Strand specific probes were developed (Table 1) and tested on the total RNA extracted from DWV infected honeybees showing clinical symptoms. In order to have a better recovery of negative strand intermediates from replicating viruses, RNA was isolated with RNeasy Lipid Tissue Mini kit. Samples from honeybees with deformed wings tested positive for the presence of the negative and positive strand (Figure 1B). No band of the correct size was obtained in the RT-free controls, for either the positive-strand or negative-strand MLPA reaction (Figure 1B), showing that the NAD-dependent ligase-65 used in RT-MLPA cannot ligate DNA probe oligonucleotides that are hybridized to RNA.

The newly developed technique, “BeeDoctor”, was used in a survey of the prevalence and distribution of the targeted viruses in Flemish apiaries. This survey revealed that almost 80% of the samples were positive for at least one of the viruses screened for by “BeeDoctor” (Table 2). No virus was detected in 21.5% of samples, 52,6% of samples had only a single virus detected, with DWV the most common virus; 23,4% of samples had double infections, with DWV-SBV the most common combination, and 2.5% of samples had 3 viruses detected. There was no regional variation in prevalence for any of the viruses. Association studies (Table 2) shows that the double, triple, fourfold and fivefold infections are totally predictable from the individual prevalences of the different viruses. The occurrence of each virus is thus independent from the other viruses and this on all virus levels.

**Table 2:** Prevalence, co-infection rates and the results of the association analysis of honeybee viruses in Flemish apiaries.

			FREQUENCY	TOTAL	ASSOCIATION
				PREVALENCE	ANALYSIS
ZERO VIRUSES	**TOTAL**	**78**	**21,5%**		n.a.
ONE VIRUS	ABPV	1	0,3%	3,3%	n.a.
	BQCV	14	3,9%	13,5%	n.a.
	CBPV	2	0,6%	1,7%	n.a.
	DWV	164	45,2%	69,4%	n.a.
	SBV	10	2,8%	19,0%	n.a.
	SBPV	0	0,0%	0,0%	n.a.
	**TOTAL**	**191**	**52,6%**		
					?^2^_(1)_
TWO VIRUSES	ABPV-BQCV	0	0,0%	-	0,06^n.s.^
	ABPV-CBPV	0	0,0%	-	0,06^n.s^
	ABPV-DWV	9	2,5%	-	0,04^n.s.^
	ABPV-SBV	1	0,3%	-	0,03^n.s.^
	BQCV-CBPV	0	0,0%	-	0,02^n.s.^
	BQCV-DWV	23	6,3%	-	1,79^n.s.^
	BQCV-SBV	5	1,4%	-	1,11^n.s.^
	CBPV-DWV	2	0,6%	-	0,00^n.s.^
	CBPV-SBV	0	0,0%	-	0,00^n.s.^
	DWV-SBV	45	12,4%	-	2,19^n.s.^
	**TOTAL**	**85**	**23,4%**		
					?^2^_(3)_
THREE VIRUSES	ABPV-BQCV-CBPV	0	0,0%	-	0,53^n.s.^
	ABPV-BQCV-DWV	0	0,0%	-	4,47^n.s.^
	ABPV-BQCV-SBV	0	0,0%	-	1,10^n.s.^
	ABPV-CBPV-DWV	1	0,3%	-	0,42^n.s.^
	ABPV-CBPV-SBV	0	0,0%	-	0,24^n.s.^
	ABPV-DWV-SBV	0	0,0%	-	6,91^P<0.10^
	BQCV-CBPV-DWV	0	0,0%	-	1,86^n.s.^
	BQCV-CBPV-SBV	0	0,0%	-	1,12^n.s.^
	BQCV-DWV-SBV	7	1,9%	-	5,33^n.s.^
	CBPV-DWV-SBV	1	0,3%	-	1,94^n.s.^
	**TOTAL**	**9**	**2,5%**		
					?^2^_(9)_
FOUR VIRUSES	ABPV-BQCV-CBPV-DWV	0	0,0%	-	3,09^n.s.^
	ABPV-BQCV-CBPV-SBV	0	0,0%	-	2,22^n.s.^
	ABPV-BQCV-DWV-SBV	0	0,0%	-	9,04^n.s.^
	ABPV-CBPV-DWV-SBV	0	0,0%	-	5,94^n.s.^
	BQCV-CBPV-DWV-SBV	0	0,0%	-	4,81^n.s.^
	**TOTAL**	**0**	**0,0%**		
					?^2^_(21)_
FIVE VIRUSES	ABPV-BQCV-CBPV-DWV-SBV	0	0,0%	-	7,32^n.s.^
	**TOTAL**	**363**	**100,0%**		

The most prevalent virus was DWV, with 69,4% of colonies screened being positive for the presence of this virus. The high occurrence of DWV in *A. mellifera* has also been reported in several other countries [45]–[47]. On the other hand, Spain had in 2006 and 2007 a very low prevalence of 18.6 and 5.9% respectively [48].

BQCV was detected in 13,5% of the colonies and is reported to have a variable prevalence in different colonies. The prevalence changes from 10 to 90% across Europe [45], [47]–[49].

SBV was present in 19% of the Flemish colonies which is high in comparison with 2% in Hungary, 1.4% in England and 1.1% in Spain [46], [48], [49]. However in France and Uruguay detection rates of respectively 86% and 100% were reported [45], [47].

CBPV was detected in only 1,7% of the samples which is in correspondence with the findings of Tentcheva et al. in France, who found a maximum frequency in colonies of 4%. These low frequency rates can be explained by the finding that CBPV might persist at undetectable levels in healthy colonies [45].

The prevalence of the virus complex ABPV, IAPV and KBV is also very low, with only 3,3% of the Flemish colonies infected. These three viruses are closely related and were detected simultaneously. The infection rate of 3,3% for the ABPV family is low as each of the viruses separately have higher prevalences in other European countries. ABPV is present in 29% of the colonies sampled in England while KBV was not detected [46]. In Spain, 13% of the colonies was infected with IAPV in 2006 and 25,7% in 2007, while KBV was very low abundant in both years (<1%) [48]. In France ABPV was present in 58% of the colonies and KBV in 17% [45].

Slow bee paralysis virus could not be detected which confirms the low natural prevalence of SBPV across a large part of Europe [19].

In conclusion, in this study we developed an RT-MLPA approach to diagnose for the most common honeybee viruses in one single procedure. We were also able to develop a strand specific assay in which we can specifically screen for the negative strand intermediate as marker for effective virus replication. The multiplex power is an enormous advantage in comparison with other well established RT-PCR approaches. This RT-MLPA diagnostic tool is easy, cost effective, allows for very high throughput analyses and above all is extremely versatile. The “BeeDoctor” can easily be expanded with probes for additional pathogens and/or markers for honeybee health and disease. Moreover, the “BeeDoctor” and RT-MLPA in general also works well even with highly degraded RNA, since it requires only very short fragments of intact RNA, since the probe-specific RT primers can partly overlap with their corresponding probe and have to be elongated by only 50 nucleotides. Proper sample preservation is often difficult to achieve in practical beekeeping and sampling in the field, and often is a limiting factor for many other screening techniques [50].

The “BeeDoctor” assay was used to screen 363 apparently healthy colonies from randomly selected apiaries throughout Flanders. This survey showed that almost 80% of colonies are infected with at least one virus, and many with multiple infections, showing that virus infections in apiaries are quite common, even in the absence of clinical symptoms.
